# Cardiorenal Tissues Express SARS-CoV-2 Entry Genes and Basigin (BSG/CD147) Increases With Age in Endothelial Cells

**DOI:** 10.1016/j.jacbts.2020.09.010

**Published:** 2020-10-09

**Authors:** Blerina Ahmetaj-Shala, Ricky Vaja, Santosh S. Atanur, Peter M. George, Nicholas S. Kirkby, Jane A. Mitchell

**Affiliations:** a Cardiorespiratory Interface, National Heart and Lung Institute, Imperial College London, London, United Kingdom; b Department of Metabolism, Digestion and Reproduction, Faculty of Medicine, Imperial College London, London, United Kingdom; c Institute of Translational Medicine and Therapeutics, Data Science Group, National Institute for Health Research, Biomedical Research Centre, Imperial College London, London, United Kingdom; d Interstitial Lung Disease Unit, National Heart and Lung Institute, Imperial College London, Royal Brompton and Harefield NHS Foundation Trust, London, United Kingdom

**Keywords:** age, cardiovascular, COVID-19, endothelial cells, ACE2, angiotensin converting enzyme 2, ADAM17, ADAM metallopeptidase domain 17, BSG, basigin, COVID-19, coronavirus disease-2019, CTSB, cathepsin B, CTSL, cathepsin L, GTEx, Genotype-Tissue Expression, PBMC, peripheral blood mononuclear cells, PPIA, peptidylprolyl isomerase A, PPIB, peptidylprolyl isomerase B, SARS-CoV-2, severe acute respiratory syndrome-coronavirus-2, TMPRSS2, transmembrane serine protease 2

## Abstract

Vascular and cardiovascular inflammation and thrombosis occur in patients with severe coronavirus disease-2019 (COVID-19). Advancing age is the most significant risk factor for severe COVID-19. Using transcriptomic databases, the authors found that: 1) cardiovascular tissues and endothelial cells express putative genes for severe acute respiratory syndrome coronavirus-2 infection, including angiotensin-converting enzyme 2 (*ACE2*) and basigin (*BSG*); 2) severe acute respiratory syndrome coronavirus-2 receptor pathways *ACE2*/transmembrane serine protease 2 and *BSG*/peptidylprolyl isomerase B(A) polarize to lung/epithelium and vessel/endothelium, respectively; 3) expression of host genes is relatively stable with age; and 4) notable exceptions are *ACE2*, which decreases with age in some tissues, and *BSG*, which increases with age in endothelial cells, suggesting that *BSG* expression in the vasculature may explain the heightened risk for severe disease with age.

## Highlights

• Cardiorenal tissues and/or endothelial cells express ACE2 and BSG
• ACE2/TMPRSS2 polarizes to lung/epithelium and BSG to vessel/endothelium
• Expression of SARS-CoV-2 host genes are mainly relatively stable with age
• Notable exceptions were; ACE2 which decreases with age in some tissues and,
• BSG which increases with age in endothelial cells

## Visual Abstract

**Figure:**
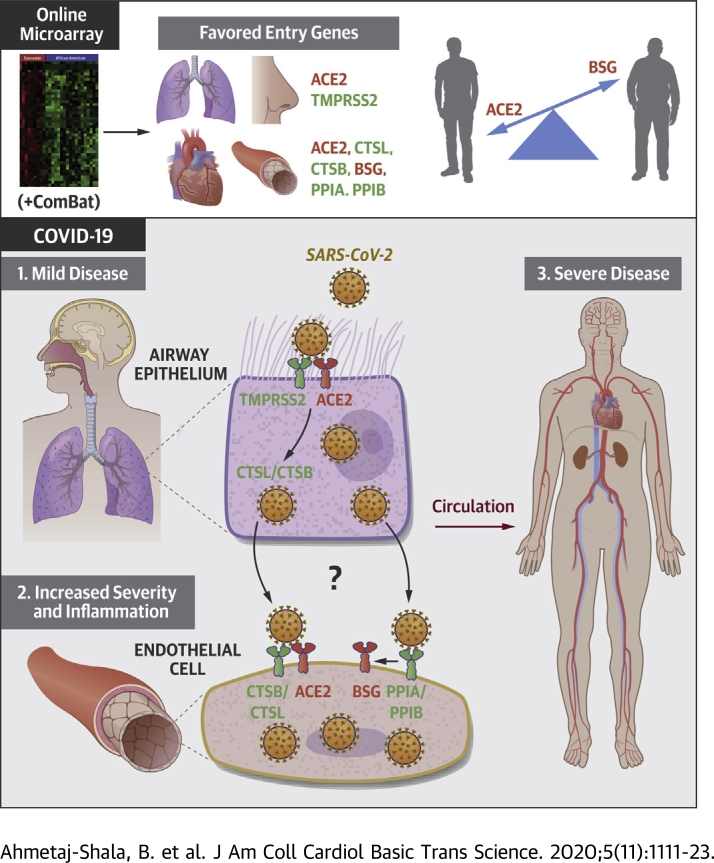

Severe acute respiratory syndrome-coronavirus-2 (SARS-CoV-2), the virus that causes coronavirus disease-2019 (COVID-19), is related to severe acute respiratory syndrome coronavirus and Middle East respiratory syndrome coronavirus, which caused respiratory epidemics in 2003 and 2012, respectively. On the basis of what was known about human host interactions with severe acute respiratory syndrome coronavirus and Middle East respiratory syndrome coronavirus along with recent research using SARS-CoV-2 tools, a list of key entry and processing genes used by the virus to infect host cells has been defined. SARS-CoV-2 enters host cells by binding of the spike protein via angiotensin-converting enzyme 2 (ACE2) (1). In addition, basigin (BSG) (also known as CD147 or EMMPRIN) is a second but putative receptor by which SARS-CoV-2 may enter cells (2,3). For viral entry by ACE2, it is thought that the SARS-CoV-2 spike protein is primed, and ACE2 cleaved, by the cellular serine proteases transmembrane serine protease 2 (TMPRSS2) (1) and ADAM metallopeptidase domain 17 (ADAM17). Intracellular processing of SARS-CoV-2 spike protein is thought to involve the lysosomal cysteine proteases cathepsin B (CTSB) and cathepsin L (CTSL), which can also substitute for TMPRSS2 in some cells (1). FURIN cleaves viral enveloping proteins, providing another putative priming step for the spike protein of SARS-COV-2 (4). For viral entry via BSG, less is known regarding specific receptor and viral processing partners for SARS-CoV-2. Indeed, firm evidence for BSG as a stand-alone receptor for SARS-CoV-2 remains the subject of investigation, with a recent study noting no “direct” binding of SARS-CoV-2 spike protein to BSG (5). However, for SARS-CoV (6), human immunodeficiency virus (7), and the measles virus (8), respectively, peptidylprolyl isomerase A (PPIA; also known as cyclophilin A) and peptidylprolyl isomerase B (PPIB; also known as cyclophilin B), which are natural ligands for BSG, incorporate into virus and facilitate binding to BSG. Similarly, cyclophilins are required for infection via BSG in malaria. In this case, PPIB forms a complex with the malaria pathogen (*Plasmodium falciparum merozoites*) and BSG to facilitate infection of red blood cells (9).

Initial infection with SARS-CoV-2 occurs via the respiratory epithelium; high gene expression of *ACE2* and *TMPRSS2* in nasal epithelium (10,11) has been taken to imply that the nose is a primary entry point for the virus (10). ACE2 and TMPRSS2 are also coexpressed in bronchial epithelium (10, 11, 12). However, where COVID-19 progresses to severe disease, the lung and other organs are also affected. The emerging pattern of severe and fatal COVID-19 includes pneumonia with acute respiratory distress syndrome, cytokine storm, widespread vasculopathy, thrombosis, renal failure, hypertension, and endothelial dysregulation seen across multiple vascular beds and organ systems (13,14). Although hypertension and thrombosis are common features after COVID-19 (13,15), the important question as to whether COVID-19 is an independent risk factor for cardiovascular disease in the acute setting and during the recovery period is a concern and remains to be established. This secondary thrombotic and vascular clinical syndrome of severe COVID-19 suggests that SARS-CoV-2 infects not only respiratory epithelium but also the endothelium, disrupting barrier function and allowing access to cardiovascular tissues and other organs of the body (16). This idea is supported by reports showing that SARS-CoV-2 can infect endothelial cells in vitro (17) and that coronaviruses including SARS-CoV-2 can progress to systemic infection (18,19), with some patients showing detectable viral ribonucleic acid in blood samples (20, 21, 22).

The reasons that underpin progression of mild to severe or fatal COVID-19 remain incompletely understood, but risk factors have been defined (23); these include established cardiovascular disease, diabetes, obesity, and black and minority ethnicity. However, the dominant risk factor for severe COVID-19 across all datasets is age, with the vast majority of those in the hospital with COVID-19 being older than 40 years. Indeed, a recent report of disparities in outcomes by Public Health England found age to be the largest disparity, with likelihood of death in adults increasing in an age-dependent manner from about 40 years (24). Importantly, although positive results for SARS-CoV-2 infection increase with age, the relative rate of infection between adult age groups profoundly underpredicts the effect of advancing age on the risk for death of COVID-19 (23,24). Therefore, understanding how SARS-CoV-2 causes severe disease with pulmonary, thrombotic, cardiorenal, and vascular complications is critically important in managing the pandemic and identifying therapeutic strategies.

Although some studies report expression profiles of *ACE2* and *TMPRSS2* in epithelial cells (10,12) and immune cells (11,12), expression patterns of a wider range of host SARS-CoV-2 entry and processing genes in these cells were recently reported (12). However, the relative expression levels of SARS-CoV-2 entry and processing genes in vessels and in endothelial cells have not been fully established. Finally, the impact of age on the expression of these genes in a cardiovascular setting is incompletely understood.

Here we have used publicly available gene expression data to determine the relative expression of key SARS-CoV-2 host entry and processing genes in human cardiorenal tissues, including aorta, coronary artery, heart (atria and left ventricle), whole blood, and the kidney and for comparison the colon, spleen, and lung. We went on to investigate gene expression in endothelial cells and, for comparison, airway (nasal and bronchial) epithelium and leukocytes (peripheral blood mononuclear cells [PBMCs]). We used blood outgrowth endothelial cells as a model because, as they are obtained from blood samples of living donors, datasets across age ranges have been created. Furthermore, blood outgrowth endothelial cells are an accepted model for application in personalized medicine, as they retain elements of disease phenotype across a number of cardiovascular and other conditions (25, 26, 27). After mapping gene expression across our target tissues and cells, our primary objective was to determine how age, as the single most dominant risk factor for severe COVID-19, affects expression of SARS-CoV-2 entry and processing genes in human cardiorenal and other tissues.

## Methods

### Ethical approval

All data were derived from publicly available open-access databases and so did not require ethical approval.

### Genotype-Tissue Expression analysis

The Genotype-Tissue Expression (GTEx) project (28) is an ongoing effort to build a comprehensive public resource to study tissue-specific gene expression. We downloaded gene expression data from GTEx version 8 (28), which contain expression data from 54 tissues from 948 donors. We identified tissues of interest on the basis of organ systems affected by severe COVID-19 and extracted expression data specifically from those tissues. Tissues were split into 2 categories: 1) cardiorenal tissues including the aorta, coronary artery, heart (atrial and appendage), left ventricle, kidney (cortex), and whole blood; and 2) “other tissues,” including lung, colon, and spleen. We performed principal-component analysis on gene expression data from each tissue of interest. We observed that the major variation in gene expression was due to type of death (Hardy score) (Supplemental Figure 1) and so corrected for this. We normalized the gene expression data for each tissue separately using ComBat-seq (29), with Hardy score as a batch. After normalization expression data were extracted for our target genes (*ACE*, *ACE2*, *ADAM17*, *BSG*, *CTSB*, *CTSL*, *FURIN*, *PPIA*, *PPIB*, and *TMPRSS2*). The following number of donors were identified for each tissue; aorta, n = 432; coronary artery, n = 240; atrial appendage, n = 429; left ventricle, n = 432; kidney cortex, n = 85; whole blood, n = 755; lung, n = 578; colon, n = 779); and spleen, n = 241). Age identifiers in GTEx are grouped by decade, as such results were analyzed on the basis of samples that associated with 20 to 29 years of age to 70 to 79 years of age. Principal-component analysis plots of raw and processed GTEx data are presented in Supplemental Figure 1.

### Gene expression dataset systematic review analysis

Using ArrayExpress and the National Center for Biotechnology Information Gene Expression Omnibus, we identified the raw datasets (.cel files) of transcriptomic gene expression profiling by microarray of healthy adult donors for blood outgrowth endothelial cells, PBMCs, and bronchial airway (obtained from bronchial brushing) and nasal (obtained nasal brushing) epithelium. We applied strict inclusion criteria: 1) only datasets that used Affymetrix Gene Chips (.cel files) were included; 2) only datasets in which individual ages are defined were included; and 3) only datasets for “untreated” cells were included. The following studies and number of donors were identified (see Supplemental Table 1): blood outgrowth endothelial cells, 3 studies with 63 donors; PBMCs, 6 studies with 84 donors; airway (bronchial) epithelium, 2 studies with 74 donors; and nasal epithelium, 3 studies with 111 donors.

### Transcriptomic expression profiling of cell data

Raw (.cel) files were imported into Partek Flow software (Partek, St. Louis, Missouri) and aligned with STAR to the human assembly (hg19) whole genome. The data were quantified to an annotation model using Ensembl Transcripts release 75 and normalized to “counts per million” and filtered to remove genes below the reliable quantitation threshold. The gene expression values from different studies were merged on the basis of gene names. To correct for batch effects, the data were normalized using the empirical Bayes model ComBat (30). The normalized expression values of our target genes (*ACE2*, *CYPA*, *CYPB*, *BSG*, *ADAM17*, *TMPRSS2*, *FURIN*, *CTSB*, and *CTSL*) were then extracted for further downstream analysis. Principal-component analysis plots of raw and processed cell data are presented in Supplemental Figure 1.

### Statistical analysis

All data were analyzed in GraphPad Prism version 8 (GraphPad Software, La Jolla, California) and are shown as individual values, mean ± SEM or mean ± SD, or median with interquartile range for samples from n = individual donors. Data were grouped according to age as <40 or ≥40 years. Data were tested for normality of distribution and analyzed using parametric (Student’s *t*-test) or nonparametric (Mann-Whitney *U* test) tests as appropriate. If significant differences between age groups were found, correlation tests were performed using Pearson (continuous cell data variables) or Spearman (ordinal tissue data variables) tests. Details of tests used are given in individual figure legends.

## Results

**Figure 1:**
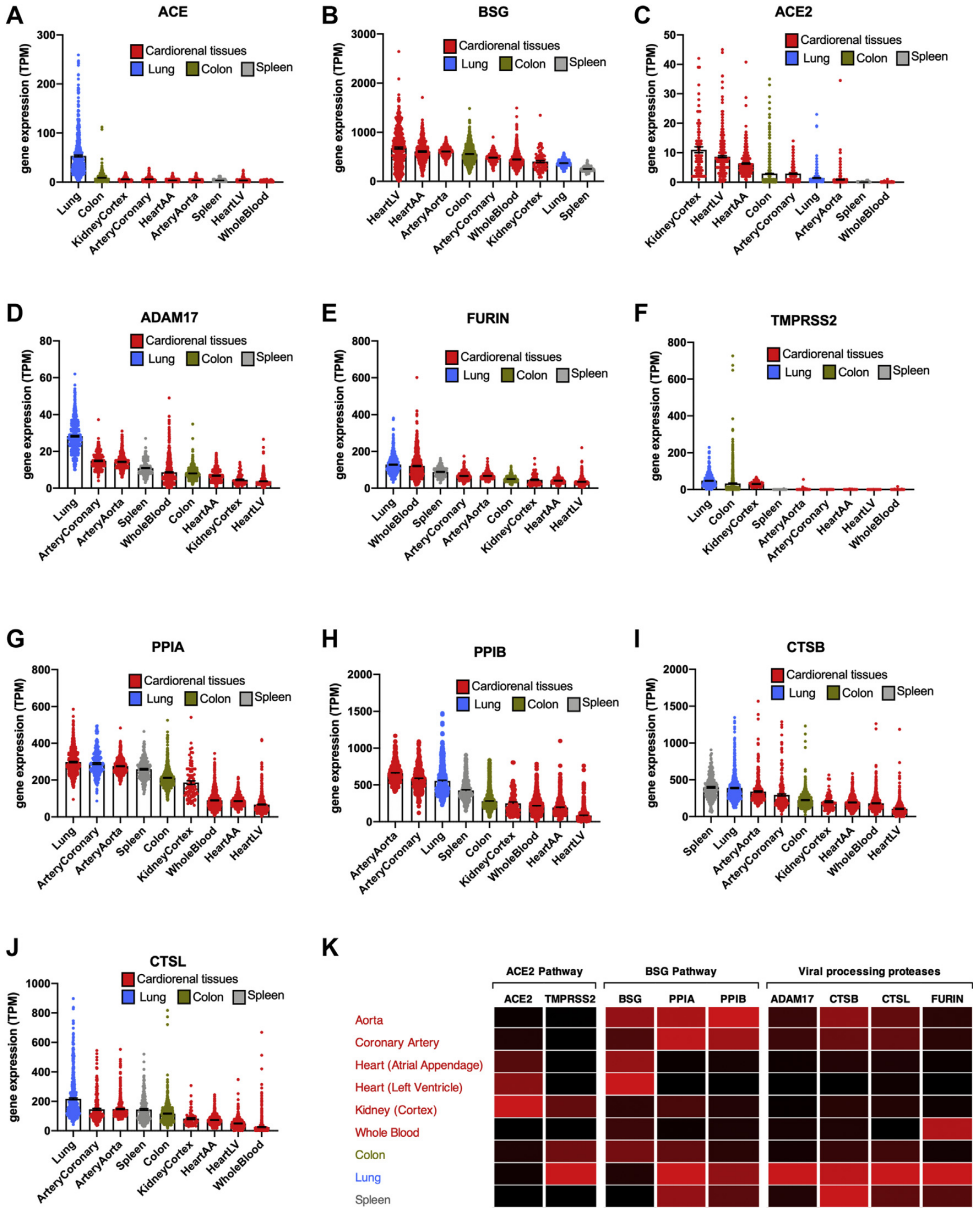
Expression of SARS-CoV-2 Entrance and Processing Genes in Different Cardiorenal and Noncardiorenal Tissues Standardized expression levels for the genes *ACE***(A)**, *BSG***(B)**, *ACE2***(C)**, *ADAM17***(D)**, *FURIN***(E)**, *TMPRSS2***(F)**, *PPIA***(G)**, *PPIB***(H)**, *CTSB***(I)**, and *CTSL***(J)** were obtained from Genotype-Tissue Expression from cardiorenal tissues (aorta, coronary artery, atrial appendage [AA], left ventricle [LV], kidney cortex, and whole blood; **red columns**) and other tissues (lung, **blue columns**; spleen, **gray columns**; and colon, **green columns**). Data for each tissue corrected for batch effects using ComBat-seq and expressed as individual points and mean ± SEM. Tissues are ranked in order of expression for each gene. A heat map showing expression of angiotensin-converting enzyme 2 (ACE2) and basigin (BSG) pathways and associated viral processing proteases in each tissue was generated **(K)**. Data are colored by gene, whereby **black** is the lowest expressing tissue and **red** is the highest expressing tissue. SARS-CoV-2 = severe acute respiratory syndrome coronavirus-2.

**Figure 2:**
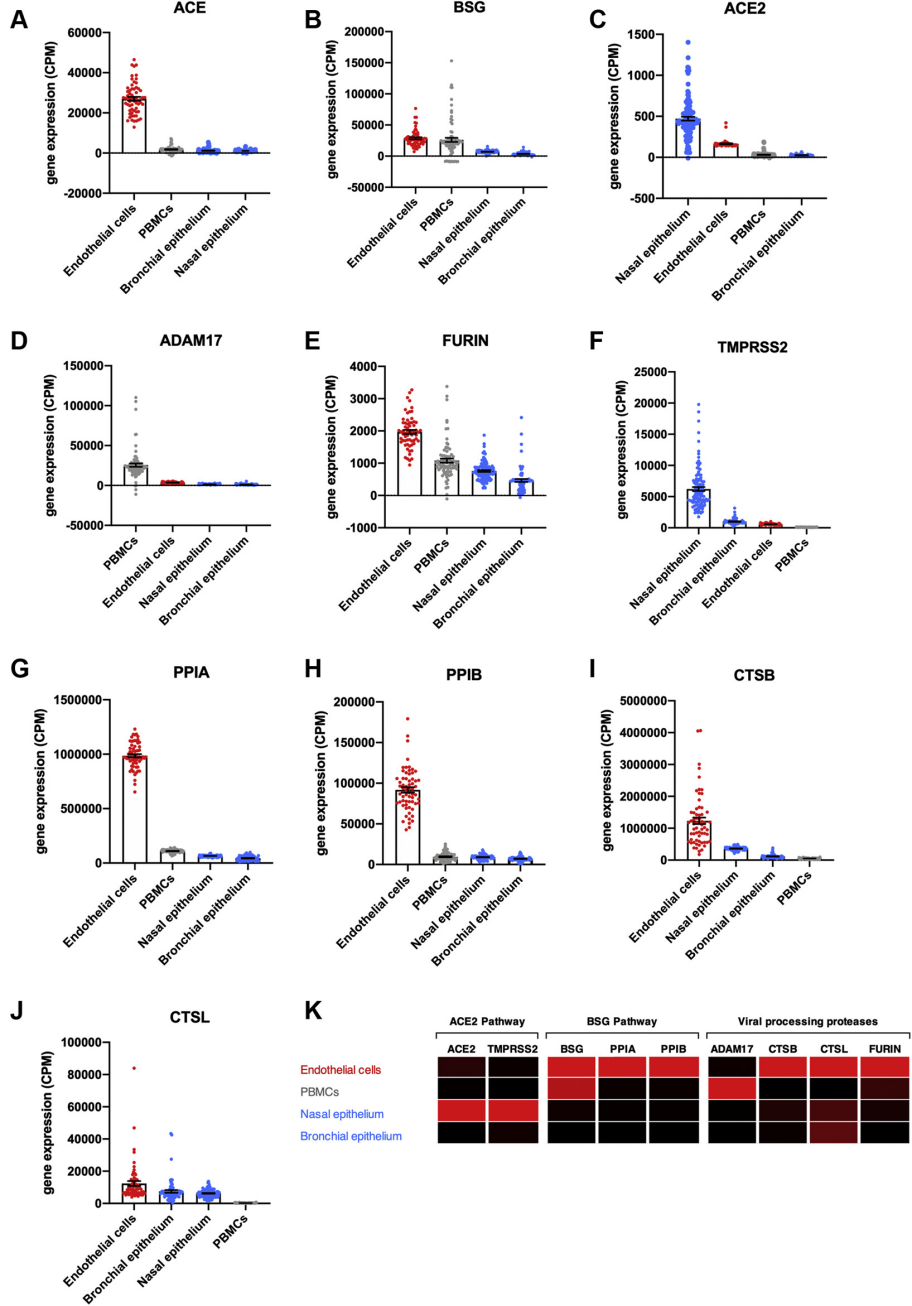
Expression of SARS-CoV-2 Entrance and Processing Genes in Blood Outgrowth Endothelial Cells, PBMCs, and Epithelial Cells (Nasal and Bronchial) Standardized expression levels for the genes *ACE***(A)**, *BSG***(B)**, *ACE2***(C)**, *ADAM17***(D)**, *FURIN***(E)**, *TMPRSS2***(F)**, *PPIA***(G)**, *PPIB***(H)**, *CTSB***(I)**, and *CTSL***(J)** were obtained from online databases from human blood outgrowth endothelial cells (endothelial cells; **red columns**), peripheral blood mononuclear cells (PBMCs) **(gray columns)**, and epithelial cells (nasal and bronchial; **blue columns**). The data were aligned and analyzed using Partek Flow and corrected for batch effects using ComBat-seq and expressed as individual data points and mean ± SEM. Cells were ranked in order of expression each gene. A heat map showing expression of ACE2 and BSG pathways and viral processing proteases in each cell type was generated **(K)**. Data are colored by gene, whereby **black** is the lowest expressing cell type and **red** is the highest expressing cell type. Abbreviations as in Figure 1.

We quantified 9 SARS-CoV-2 entry and processing genes (*ACE2*, *BSG*, *ADAM17*, *TMTRSS2*, *PPIA*, *PPIB*, *CTSB*, *CTSL*, and *FURIN*) (Figures 1 and 2) along with *ACE* in cardiorenal tissues (including kidney and whole blood) and other organs (including lung spleen and colon) (Figure 1) and in endothelial cells, respiratory epithelial cells, and PBMCs (Figure 2). ACE is not directly related to cellular processing of the virus but represents a pharmacological link with ACE2.

### Relative expression of SARS-CoV-2 entry genes across organs

**Figure 3:**
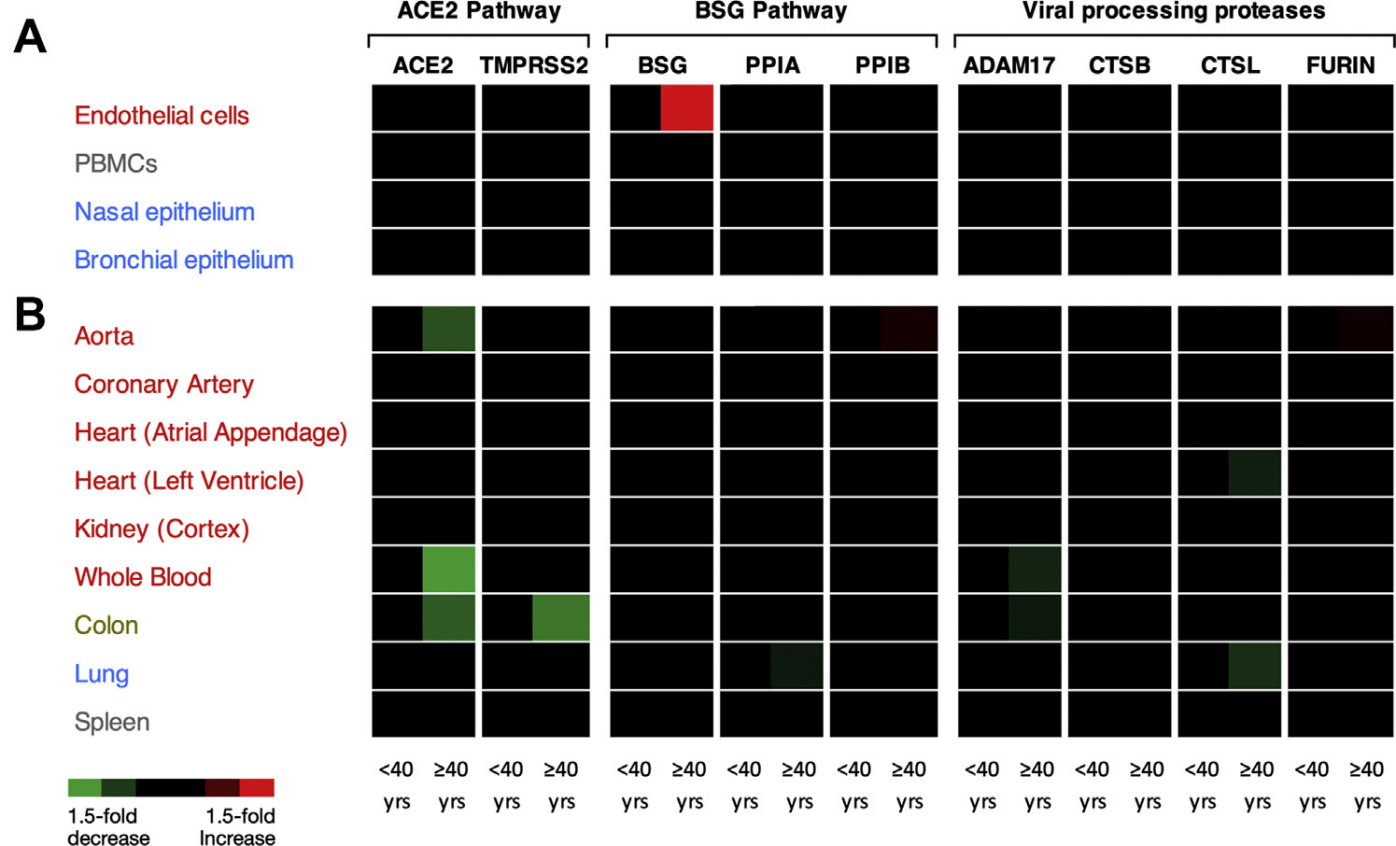
Heat Map Representing Expression of SARS-CoV-2 Entrance and Processing Genes Significantly Altered by Age Heat maps were generated for the expression of *ACE*, *ACE2*, *BSG*, *ADAM17*, *CTSB*, *CTSL*, *FURIN*, *PPIA*, *PPIB*, and *TMPRSS2* in cells **(A)** and organs **(B)**. Data were analyzed on the basis of 2 adult age groups; <40 or >40 years. Data were analyzed using an unpaired Mann-Whitney *U* test or Student’s *t*-test depending on normality. Significant data (p < 0.05) are shown as either increased **(red)** or decreased **(green)** expression; **black** corresponds to no significant change.

As expected, *ACE* was highly expressed in the lung (31) with lower but consistent levels expressed across other tissues and with very low levels present in blood (Figures 1 and 3). Of the 2 putative SARS-CoV-2 receptors, *BSG* was highly expressed across all tissues, with higher levels seen in most cardiorenal tissues than in the lung or spleen. *ACE2*, across all tissues, was expressed in relatively low levels. However, cardiorenal tissues including kidney, heart, and blood vessel (coronary artery) expressed higher levels of *ACE2* than the lung or spleen. Relatively low levels of *ACE2* were seen in whole blood. The colon was positioned midgroup for both *BSG* and *ACE2* expression. Of the putative processing genes, required for spike protein conditioning and/or cleavage of *ACE2* allowing viral entry, *ADAM17* and *FURIN* were each enriched in the lung with relatively stable levels of expression across cardiorenal and other target tissues. *TMPRSS2* was also enriched in the lung, colon, and kidney cortex with very low levels present in arteries, heart, spleen, and blood. For the putative vial partner ligands of *BSG*, *PPIA*, and *PPIB*, both were expressed throughout our selected tissues, with higher levels expressed in arteries than kidney, blood, or heart tissues. Lung, spleen, and colon expressed high or midranking levels of *PPIA* and *PPIB*. The endosomal proteases *CTSB* and *CTSL* showed similar expression patterns across our tissues of interest. Both *CTSB* and *CTSL* were enriched in the lung and spleen (*CTSB*), with relatively high levels across all tissues. *CTSB* and *CTSL* were more highly expressed in arteries than in kidney, heart, or blood.

### Relative expression of SARS-CoV-2 entry genes in endothelial cells versus airway epithelium and PBMCs

To complement and extend the aforementioned organ-level approach, we focused on endothelial cells versus respiratory epithelial cell types and immune cells (PBMCs), in line with COVID-19 pathology (Figures 2 and 3). As expected, *ACE* was highly enriched in endothelial cells (32), with lower levels present in PBMCs and bronchial and nasal epithelium. *BSG* was enriched in endothelial cells and PBMCs, with lower levels expressed in nasal and bronchial airway epithelium. *ACE2* was enriched in nasal epithelium, followed by endothelial cells and lower levels in PBMCs and bronchial epithelium. *ADAM17* was highly enriched in PBMCs, followed by endothelial cells and nasal and bronchial epithelial cells. *FURIN* was enriched in endothelial cells with midranking levels expressed in PBMCs and lower levels in nasal and bronchial epithelium; *TMPRSS2* was highly enriched in nasal epithelial cells, followed by bronchial epithelial cells, endothelial cells, and PBMCs. *PPIA* and *PPIB* were highly expressed in endothelial cells with lower levels in PBMCs and nasal and bronchial epithelial cells. Intracellular proteases *CTSB* and *CTSL* were both also enriched in endothelial cells, followed by airway epithelium (*CTSB*, nasal > bronchial; *CTSL*, bronchial > nasal) and low levels in PBMCs.

Next, in line with Public Health England’s recent review of disparities in risks and outcomes for COVID-19 (11), we grouped data into 2 age categories, <40 and >40 years, to determine differences in gene expression. Where differences were found and on the basis of clinical evidence showing that the risk for death of COVID-19 directly correlates with age (13), we performed follow-on correlation analysis.

### Effect of age on expression of SARS-CoV-2 entry genes across cardiorenal and COVID-19 target tissues

#### Arteries

**Figure 4:**
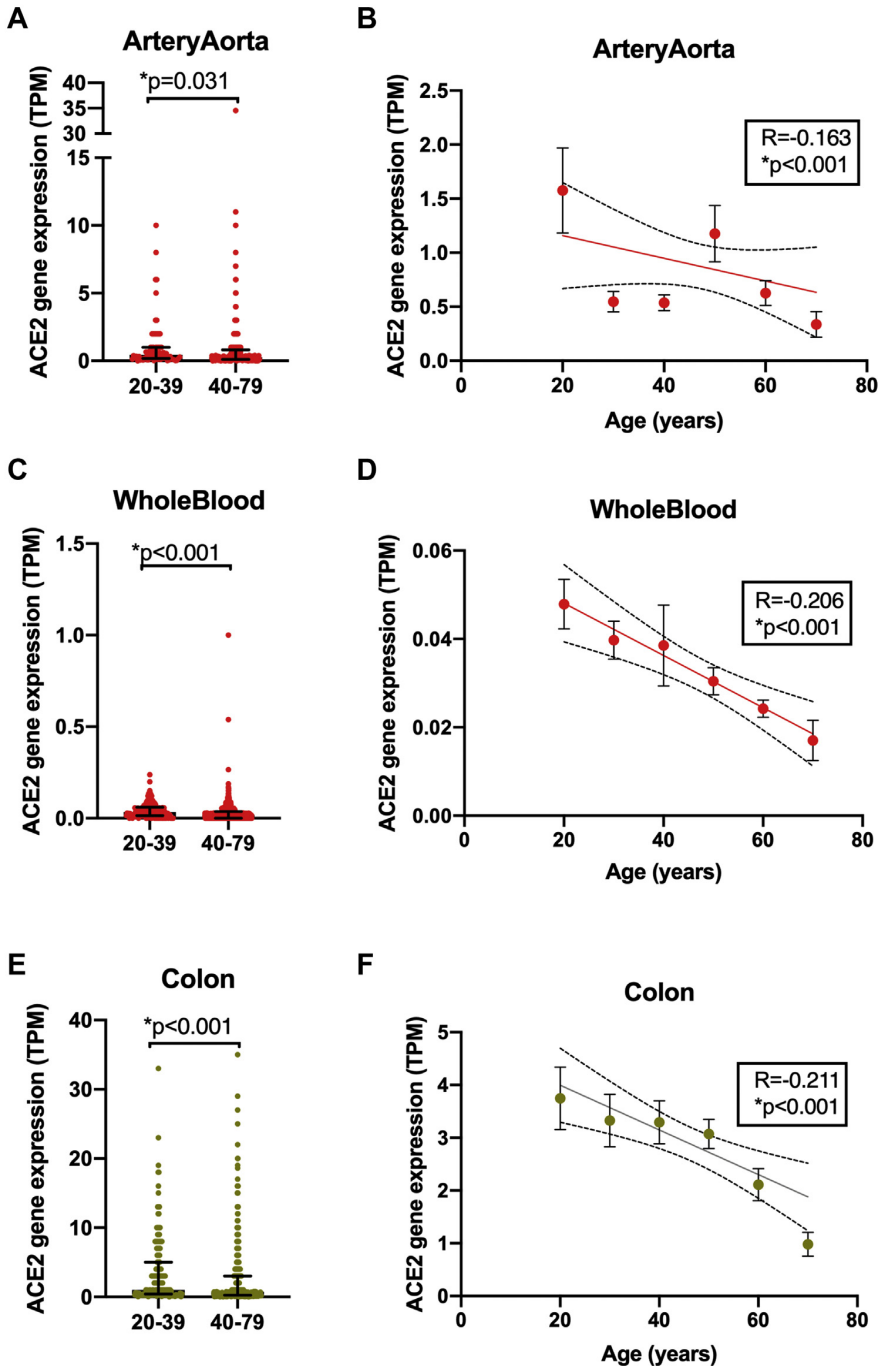
The Effect of Age on *ACE2* Expression in Aorta, Whole Blood, and Colon *ACE2* levels in aorta **(A,B)**, whole blood **(C,D)**, and colon **(E,F)** were analyzed on the basis of adults <40 versus >40 years of age **(A,C,E)**. Data were analyzed using unpaired Mann-Whitney *U* test *(A,C,E)* or Spearman correlation test **(B,D,F)**. Data are expressed as individual points and median ± interquartile range **(A,C,E)** or mean ± SEM **(B,D,F)**; significance was accepted for ∗p < 0.05.

In the aorta, *FURIN* and *PPIB* were increased while *ACE2* was deceased in samples from adults >40 years of age (Figure 3). Reductions of *ACE2* or increases of *PPIB* linearly correlated with age (Figure 4, Supplemental Figure 2). *FURIN* expression did not linearly correlate with age (Supplemental Figure 2). None of our selected genes were affected by age in the coronary artery (Figure 3).

#### Heart and kidney

In the heart (atria and left ventricle) or kidney, none of our selected genes were affected by age (Figure 3, Supplemental Figure 2).

#### Whole blood

In whole blood, *ACE* expression increased, while *ACE2* and *ADAM17* were reduced in samples from subjects >40 years of age (Figures 3 and 4, Supplemental Figure 2). Of these genes, reduced expression of *ACE2* (Figure 4) and *ADAM17* and increased expression of *ACE* linearly correlated with age (Supplemental Figure 2).

#### Colon

In the colon, *ACE2*, *ADAM17*, and *TMPRSS2* were decreased in samples from subjects >40 years of age (Figures 3 and 4, Supplemental Figure 3). Of these genes, reduced expression levels of *ACE2* and *TMPRSS2* but not *ADAM17* linearly correlated with age (Supplemental Figure 3).

#### Lung

In the lung, expression levels of *CTSL* and *PPIA* were decreased in samples from adults >40 years of age (Figure 3). Reduced expression linearly correlated with age for *PPIA* but not *CTSL* (Supplemental Figure 3).

#### Spleen

None of our studied genes were altered with age in the spleen (Figure 3).

### Effect of age on expression of SARS-CoV-2 entry genes in endothelial cells, airway cells, and leukocytes

**Figure 5:**
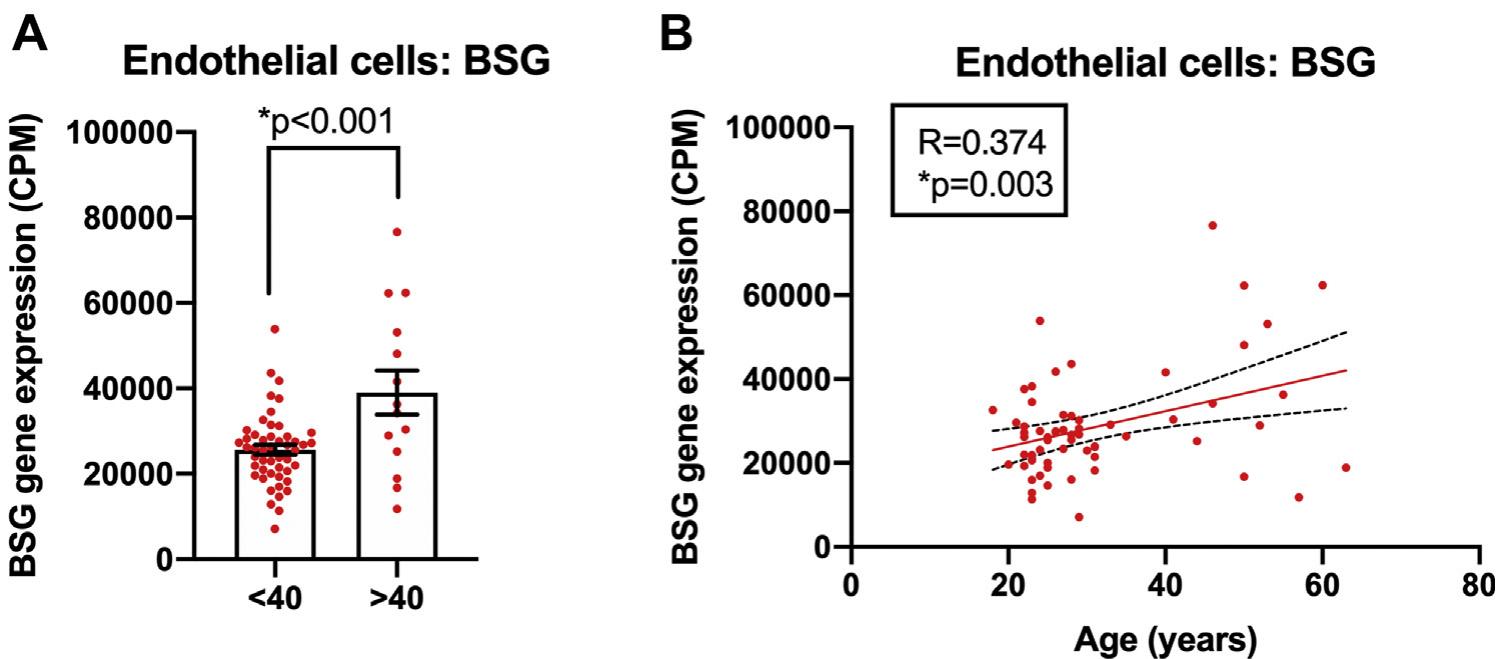
The Effect of Age on *BSG* Expression in Blood Outgrowth Endothelial Cells *BSG* levels in blood outgrowth endothelial cells (endothelial cells) were analyzed in adults <40 years of age versus ≥40 years of age using an unpaired Student’s *t*-test **(A)** and correlations with age determined using Pearson correlation analysis **(B)**. Data are expressed as individual data points and mean ± SEM; significance was accepted at ∗p < 0.05.

In endothelial cells *BSG*, but not other genes, was increased in samples from adults >40 years of age (Figures 3 and 5), and levels showed a positive linear correlation with age (Figure 5). In contrast, only *ACE* was fractionally (but statistically significantly) reduced in nasal epithelium between age categories (Figure 3), and this did not linearly correlate with age (Supplemental Figure 4). No genes were altered in bronchial epithelium and PBMCs with age (Figure 3).

Although not the primary outcome of our study, which was age, we also investigated the effect of sex of the expression of key SARS-CoV-2 genes. In cells we noted the following changes; men had significantly increased gene expression of *CTSB*, *BSG*, *PPIA*, and *PPIB* in endothelial cells, bronchial epithelium, and nasal epithelium, respectively (Supplemental Figure 5). Men had reduced levels of *PPIB* and *ADAM17* in endothelial cells and nasal epithelium, respectively (Supplemental Figure 5). In tissues we noted the following changes: men had significantly higher levels of *ACE* and *TMPRSS2* gene expression in the heart atrial appendage and coronary artery, respectively. In contrast, there was a significant reduction in the gene expression of *TMPRSS2*, *ACE2*, and *BSG* i32n the heart left ventricle and whole blood, respectively (Supplemental Figure 5).

### Summary

In light of the cardiovascular and thrombotic sequalae associated with severe COVID-19 and the overwhelming risk that increased age carries, our aim was to obtain mechanistic insight by interrogating gene expression profiles in cardiovascular tissues and cells. Our focus was on the SARS-CoV-2 receptor ACE2 and the putative receptor pathway BSG, along with a selected range of genes thought to be involved in virus binding and processing. In this study we have made 4 important observations: 1) cardiorenal tissues and/or endothelial cells express the required genes for SARS-CoV-2 infection, including *ACE2* and *BSG*; 2) ACE2/TMPRSS2 and BSG/PPIB(A) somewhat polarize to lung/epithelium and vessel/endothelium, respectively; 3) expression of SARS-CoV-2 host genes is, on the whole, relatively stable with age; and 4) notable exceptions were *ACE2*, which decreases with age in some tissues, and *BSG*, which increases with age in endothelial cells.

## Discussion

Initial SARS-CoV-2 infection occurs in the airways. For most people infection is either asymptomatic or associated with mild symptoms consistent with localized viral infection in respiratory tissues. Naturally, therefore, most research to date has focused on investigations in the lung or airway cells and understanding how to manage the complications of pneumonia and ventilation failure. However, in some patients, SARS-CoV-2 infection progresses to severe COVID-19, which is a systemic illness with complications specifically associated with the cardiorenal system, endothelium, and thrombosis. Now, understanding the vascular component of severe COVID-19 associated with SARS-CoV-2 infection is emerging as an urgent unmet clinical need.

In this paper, we report that SARS-CoV-2 receptors and processing genes are expressed across all cardiorenal target tissues and/or in endothelial cells, supporting the idea that systemic organs contain the required machinery to be infected by the virus. With regard to the 2 SARS-CoV-2 receptor pathways, expression levels of *ACE2* were higher in cardiovascular tissues than the lung. IN contrast, *TMPRSS2*, thought to be required for SARS-CoV-2 infection in epithelial cells, was present in lung, colon, and kidney but essentially absent in other cardiovascular tissues (heart, vessels, and whole blood). Our findings describing the relative levels of these genes in human tissues are in line with others using similar approaches for *ACE2* (32, 33, 34, 35, 36) and *TMPRSS2* (35,37). Moreover, we found that both *ACE2* and *TMPRSS2* were enriched in nasal epithelium, with low levels in bronchial epithelium and PBMCs, which is in agreement with recent work from others (nasal vs. bronchial epithelium [10,11] and versus PBMCs [11]). Radzikowska et al. (12) profiled a wider range of SARS-CoV-2 entry genes in immune cells and differentiated primary bronchial epithelial cells and also reported relatively high levels of expression of *PPIA*, *BSG*, and *PPIB*, with much lower levels of *TMPRSS2* followed by *ACE2* in airway cells. However, our focus was on the cardiovascular system and kidney. Importantly, we confirm that endothelial cells express *ACE2* and *TMPRSS2*, although at lower levels than nasal epithelium but higher levels than bronchial epithelial cells (*ACE2*) and PBMCs (*ACE2* and *TMPRSS2*). These findings suggest that SARS-COV-2 could infect endothelial cells via the ACE2 pathway. In cells in which TMPRSS2 is low, SARS-CoV-2 can gain access by using CTSL and/or CTSB (1). In our study both *CTSL* and *CTSB* were found to be enriched in endothelial cells. Furthermore, in the setting of SARS-CoV-2 infection, this pathway may well be facilitated by the release of lysosomal proteases during inflammation (38).

Nevertheless, in contrast to the ACE:TMPRSS2 pathway, which, on the basis of *TMPRSS2* expression levels, was better represented in lung and nasal epithelium, the BSG:PPIB/PPIA pathway was, as a whole, better represented in vessels and endothelial cells than in the lung and airway epithelial cells. Our findings suggest that SARS-CoV-2 and other relevant viruses may, in addition to ACE2, exploit BSG as receptor pathway in the vasculature. Our findings align with recent work by Ganier et al. (39) and add evidence to the recent “proposed mechanism” explaining how SARS-CoV-2 accesses endothelial cells, presented by Acosta Saltos and Acosta Saltos (40).

Severe COVID-19 is exceptionally rare in children. In adults, the strongest risk factor for severe disease and death is age, with those younger than 40 years being at very low risk; the risk for severe COVID-19 and death increases proportionally after the age of 40 (24). Of the genes we studied, several candidates, including *ACE2*, were affected by age, but with the exception of *BSG* in endothelial cells and *PPIB* and *FURIN* in aorta, expression was reduced in those >40 years of age. We found consistent age-related reductions in *ACE2* in whole blood, aorta, and the colon. Our findings are in line with those published by Chen et al. (33), who also reported a negative correlation between *ACE2* and age in a range of tissues including colon and blood. Moreover, our work corroborates earlier studies showing that ACE2 (protein) declines with age in mouse aorta (41). Other studies in rats also showed that ACE2 declines with age in the lung and kidney (42,43). It should be noted, however, that Chen et al. (34) found no effect of age on *ACE2* expression across a similar selection of tissues and that Santesmasses et al. (44) found that *ACE2* expression increased with age in the lung. We also found a trend for *ACE2* to increase in the lung, but this did not reach statistical significance in our study. Key differences between the studies include the analytic approaches applied, the number of tissues selected, and the age groups used.

As ACE2 is a receptor for SARS-CoV-2, which declines with age in some settings (this study) (34,41, 42, 43), and because age is the strongest predictor of fatal COVID-19, a paradox has emerged (45): SARS-CoV-2 receptor expression does not positively correlate with high-risk groups of severe COVID-19. One explanation for the paradox has been that because ACE2 is a cardioprotective enzyme, while ACE2 is the receptor for airway infection of SARS-CoV-2, low levels of ACE2 in the circulation of elderly patients and those with cardiovascular disease increase the risk for cardiovascular complications associated with severe COVID-19 (45). Additionally, it has been hypothesized that BSG acts as a receptor for SARS-CoV-2 in endothelial cells (40). BSG expression is increased in a range of cardiovascular diseases, which could compensate for any age- or disease-associated reductions in ACE2 in regard to viral infection. In line with this in our study, *BSG* positively correlated with age, and this association was seen only in endothelial cells. Others have found that BSG increases with age in the skin (46). It is not clear why we did not see age-related increases in *BSG* in the aorta or coronary artery or in organ samples. One explanation could be that in vessels, the delicate lining of the endothelium may have been lost during tissue dissection and/or that the age effects on *BSG* expression in endothelial cells are diluted out in complex tissues by expression levels in other cells that make up the bulk of the samples. It should also be considered that although blood outgrowth endothelial cells display key ubiquitous features of endothelial cells and retain disease phenotypes (25), heterogeneity exists within endothelial cells as they age with passage and in different vascular beds.

On the basis of our findings, we suggest that BSG expression in the vasculature maybe an important driver that explains the heightened risk for severe disease and death observed in those >40 years of age with severe COVID-19. These observations add to the growing evidence and provide additional mechanistic insight supporting the targeting the BSG: PPIA/PPIB axis in severe COVID-19.

BSG expression data in endothelial cells increasing with age can be viewed as supporting a possible role of CD147 in CoV-2 binding/entry, with more work needing to be done. Specifically, it is important to further assess susceptibility and permissibility of endothelial cells to SARS-CoV-2 and the role (if any) BSG plays in infection. Nonetheless, BSG is up-regulated in a range of diseases, including those comorbidities or morbidities associated with increased risk for severe COVID-19 disease and poorer outcomes including thrombosis (47), pulmonary hypertension (48), renal disease (49), obesity (12), and diabetes (50). Moreover, BSG, as an inducer of extracellular matrix metalloproteinases, may be relevant when considering the potential role of the vasculature, specifically endothelial dysfunction, in propagating and driving pulmonary fibrosis (51,52), 1 of the most feared long-term complications of COVID-19 (53).

Clearly, a full understanding of BSG interactions with SARS-CoV-2 will provide valuable mechanistic insight and could identify new therapeutic targets and/or provide additional insight for experimental drugs currently in trials for the prevention and treatment of severe COVID-19. In light of our findings and the overwhelming clinical information indicating vascular inflammation in severe COVID-19, the relative role of SARS-CoV-2 receptors and processing proteins in endothelial cells should be investigated. Our data indicate that blood outgrowth endothelial cells will be a useful tool in future work exploring mechanisms of viral infection and inflammation in COVID-19. In addition, as blood outgrowth endothelial cells can be obtained from blood samples of living donors, functional assays using these cells from protected and at-risk populations may provide a means of identifying personalized therapies and those at risk for severe disease.

As BSG is used by other pathogens, our findings have implications beyond the current pandemic. Finally, because BSG is implicated in a range of cardiovascular diseases and fibrosis, our observations may have relevance to our understanding of the diseases associated with aging.

### Study limitations and future studies

The details of the cellular events involved in SARS-CoV-2 infection in different cell types have not yet been fully established, which means that the weighted relevance of genes we have investigated in COVID-19 remains to be elucidated. Nevertheless, each of the genes we studied is established in various aspects of human physiology and pathology, so our analysis has relevance to the understanding of aging in a setting wider than infection. We used publicly available datasets that, although they were analyzed in a systematic and unbiased manner, require replication and validation in a prospective follow-up clinical and mechanistic studies. Furthermore, gene expression data invariably require biological validation in functional assays.

Perspectives **Competency in Medical Knowledge**

Our findings that cardiorenal tissues and endothelial cells express higher or comparable levels of SARS- CoV-2-associated genes as found in lung or airway epithelium support the idea that SARS-CoV-2 may infect the vasculature. Moreover, our data regarding viral entry and processing genes suggests that any viral infection in endothelial cells may occur via different routes to nasal epithelium, which is thought to use ACE2 and TMPRSS2, at 2 key levels. First, where SARS-CoV-2 may use ACE2 to infect endothelium, lysosomal proteases CTSL and CTSB, rather than TMPRSS2, may facilitate viral entry; second, the BSG/ PPIA/PPIB is dominate in endothelial cells compared with airway epithelium.

**Translational Outlook**

Although the role of BSG in SARS-CoV-2 infection remains to be fully established, our findings that BSG increases with age in blood outgrowth endothelial cells provide a strong translational impetus for using these cells as potential bio- markers to identify subjects or groups most at risk for vascular complications of COVID-19. In a wider setting, considering the well-established role of BSG in cardiovascular disease, our results have clear im- plications for our understanding of aging in the cardiovascular system.

## Author Disclosures

This work was funded by the Wellcome Trust/Imperial College Institutional Support Fellowship for Dr. Ahmetaj-Shala. Dr. Kirkby is a recipient of an Intermediate Research Fellowship from the British Heart Foundation (FS/16/1/31699). Dr. Vaja is a recipient of a Clinical Training Fellowship from the British Heart Foundation (FS/19/6/34129). Drs. Kirkby and Mitchell are holders of a program grant from the British Heart Foundation (RG/18/4/33541). All other authors have reported that they have no relationships relevant to the contents of this paper to disclose.
