# Supplementary material for: Cardiorenal Tissues Express SARS-CoV-2 Entry Genes and Basigin (BSG/CD147) Increases With Age in Endothelial Cells
Source: JACC Basic Transl Sci. 2020 Oct 9;5(11):1111–23. doi: 10.1016/j.jacbts.2020.09.010 (PMC7546186; doi:10.1016/j.jacbts.2020.09.010)
Supplement: Supplemental Figures 1–5 and Supplemental Table 1 [file mmc1.pdf]

SUPPLEMENTAL MATERIAL

Supplementary Figure 1. Principle component analysis (PCA) of genes of interest pre and post ComBat-Seq normalization. PCA plots for all organs (A) and cells (B) pre and post ComBat-Seq normalization were generated.

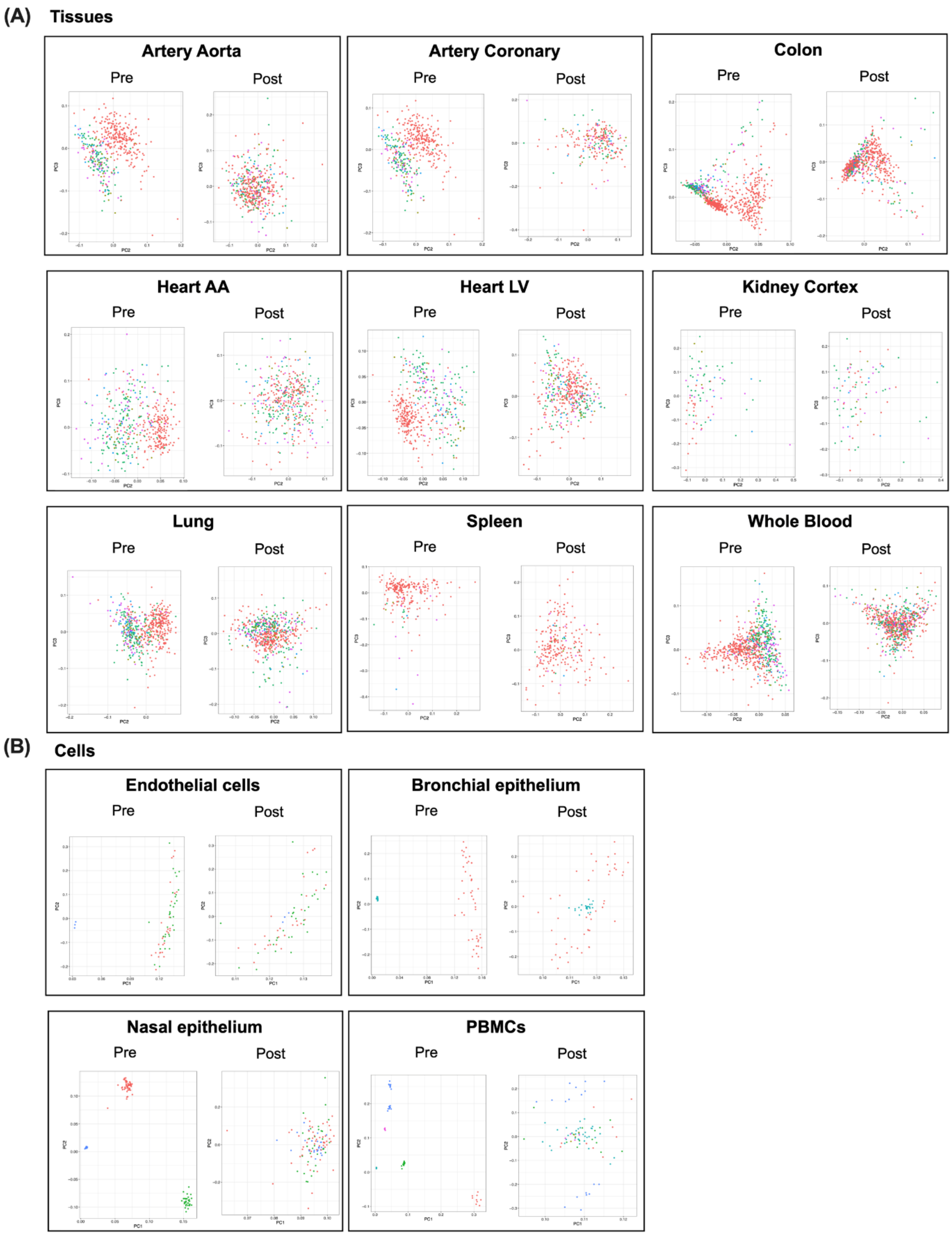

Supplementary Figure 1

Supplementary Figure 2. The effect of age on *FURIN*, *PPIPB, CTSL, ACE or ADAM17* in aorta, heart left ventricle (heartLV) or whole blood

Gene expression levels in aorta, heart left ventricle and whole blood were analyzed based on adults under 40 years (<40) versus over 40 years (≥40). Data were analyzed using an unpaired Mann-Whitney T-test (**A**, **C, E, G and I**). Correlations with age were determined using Spearman's correlation test analysis (**B**, **D, F, H and J**). Data were expressed as individual data points and median +/- interquartile range (Q1, Q3); significance was accepted when *p<0.05.

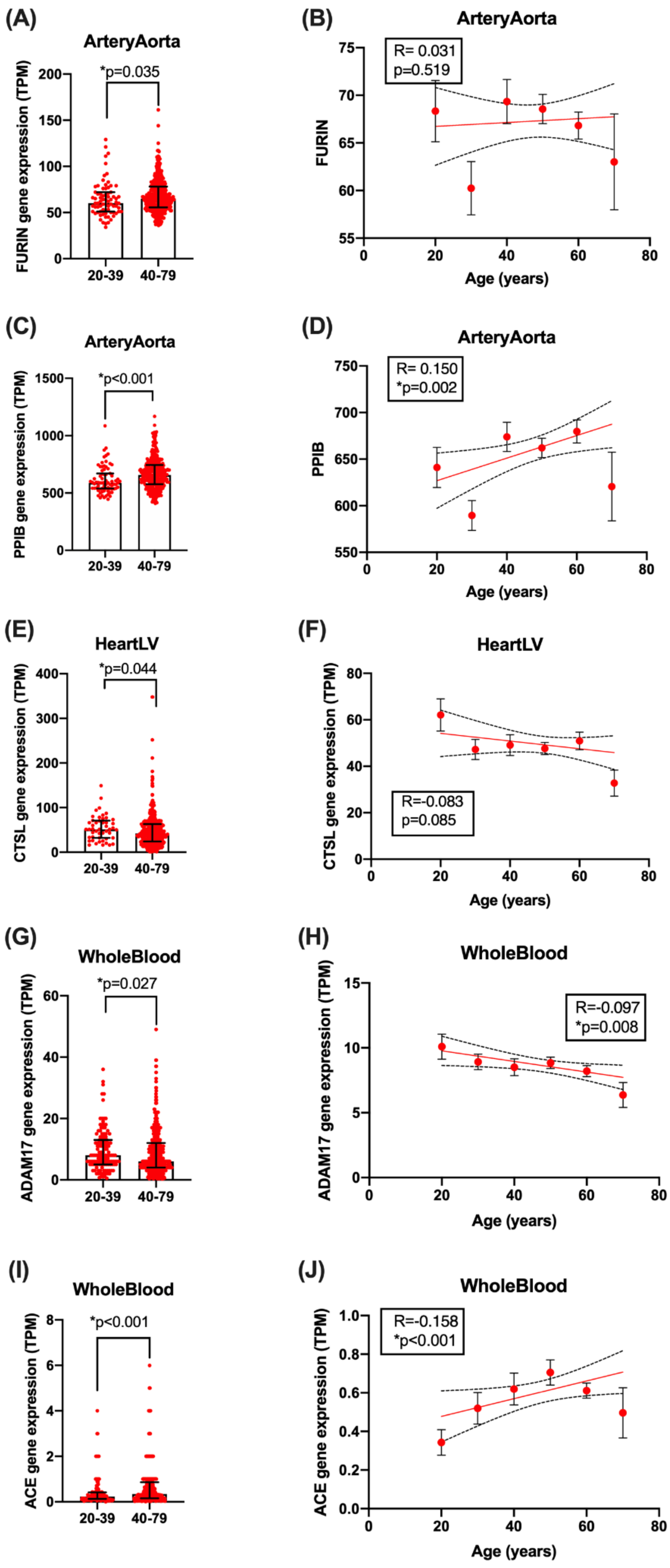

Supplementary Figure 2

Supplementary Figure 3. The effect of age on *ADAM17, TMPRSS2, CTSL* or *PPIA* in colon or lung

Gene expression levels in lung were analyzed in adults under 40 years (<40) versus over 40 years (≥40) using an unpaired Mann-Whitney T-test (**A**, **C, E, G**) and correlations with age determined using Spearman's correlation test analysis (**B**, **D, F, H**). Data were expressed as individual data points and median +/- interquartile range (Q1, Q3) (**A, C, E, G**) or mean +/- SEM (**B, D, F, H**); significance was accepted when *p<0.05.

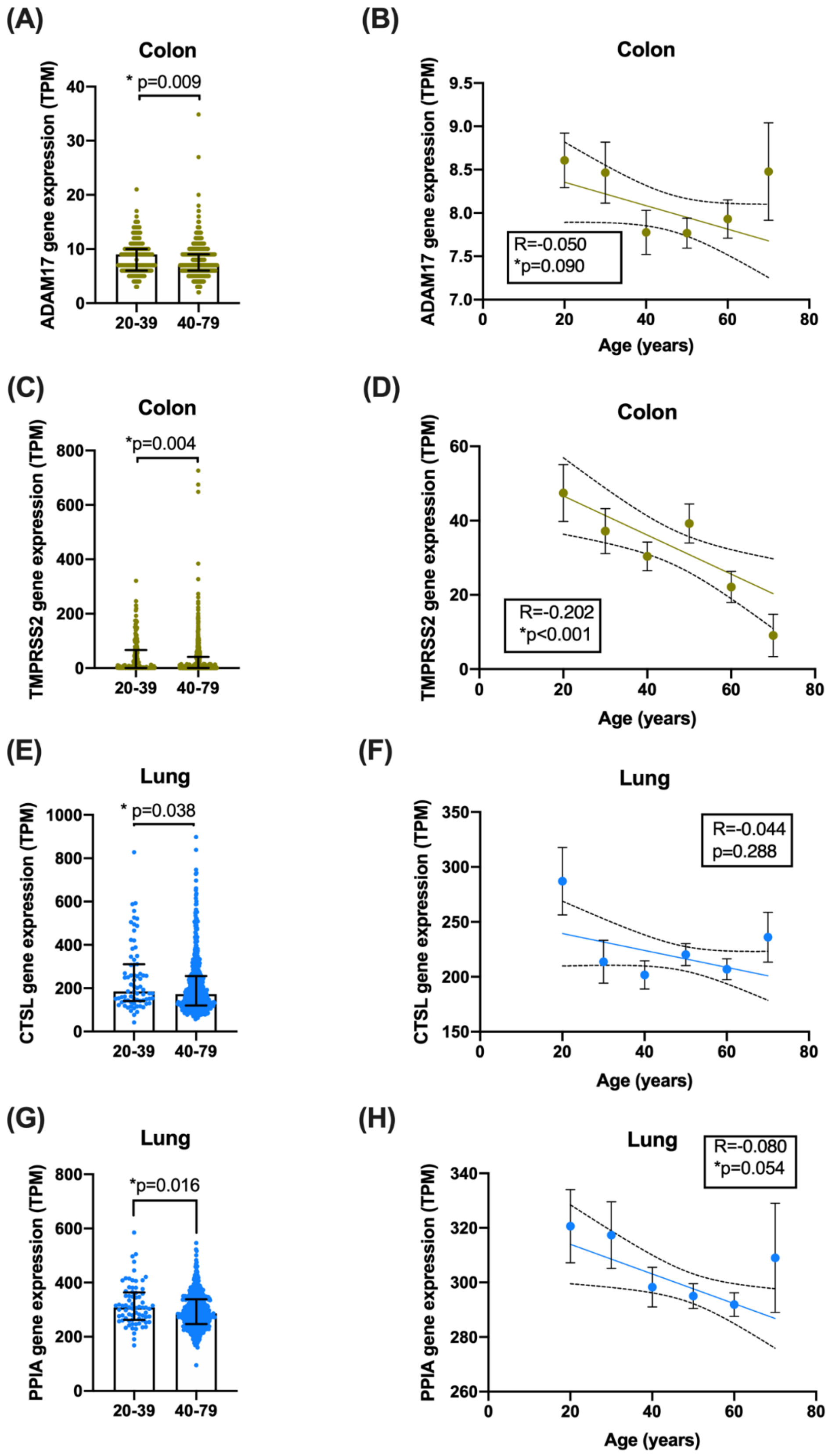

Supplementary Figure 3

Supplementary Figure 4. Correlation analysis of ACE expression in nasal epithelial cells with age

Data are shown as individual points for *ACE* expression levels and age (years). Data were expressed as individual points and were analyzed using Pearson's correlation analysis.

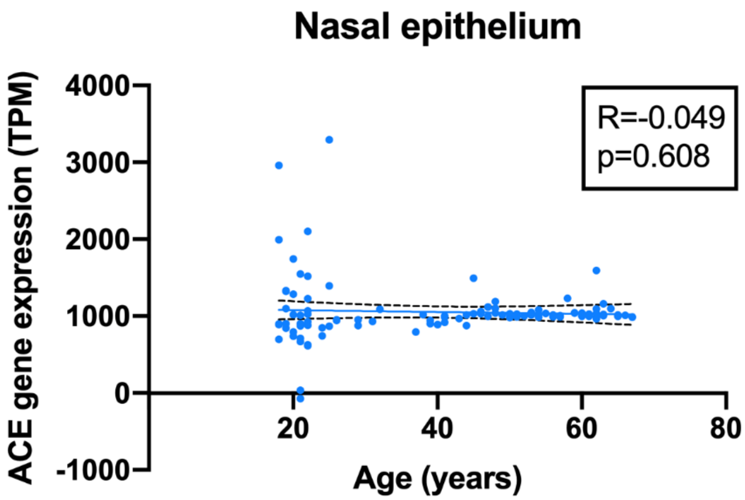

Supplementary Figure 4

Supplementary Figure 5. The effect of gender on endothelial cells, bronchial and nasal epithelium, heart appendage (heartAA), heart left ventricle (heartLV), whole blood and coronary artery. Gene expression levels in cells and tissues were analyzed in males and females using an unpaired Student's t-test (**A, B, C, D, F**) or Mann-Whitney T-test (**G, H, I, J, K**). Data were expressed as individual data points and mean +/- SEM (**A, B, C, D, F**) or median +/- interquartile range (Q1, Q3) (**G, H, I, J, K**); significance was accepted when *p<0.05.

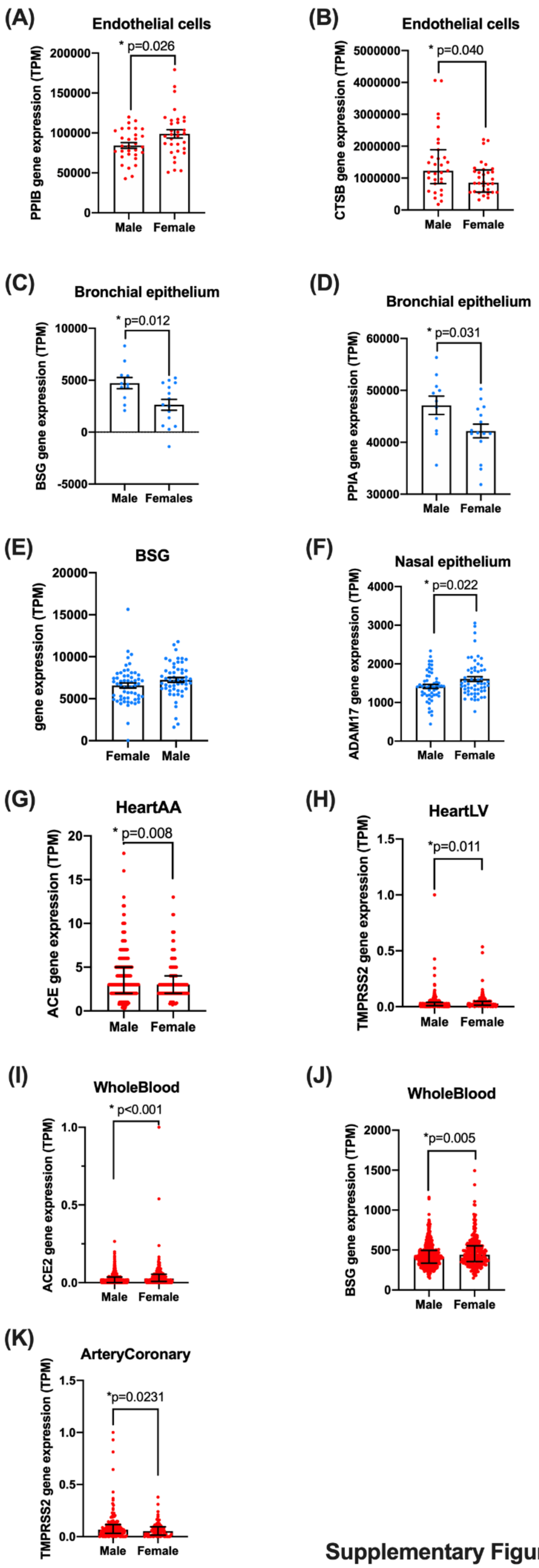

Supplementary Figure 5

Supplementary Table 1. Identified cell studies. Raw transcriptomic gene expression profiling datasets were obtained from ArrayExpress and NCBI GEO for blood outgrowth endothelial cells (BOECs; also known as 'late outgrowth endothelial cells'), peripheral blood mononuclear cells (PBMCs) and bronchial airway (obtained from bronchial brushing) and nasal (obtained nasal brushing) epithelium.

Cell type	Study ID	n	Gender (Male; % total)	Age (<40; % total)	Age (mean ± SD)	Data source weblink
**BOECs** (late outgrowth endothelial cells)	GEOD9877	27	12 (44%)	18 (67%)	38.1 ±12.8	https://www.ebi.ac.uk/arrayexpress/experiments/E-GEOD-9877/
	GEOD22688	33	18 (55%)	32 (97%)	25.0 ±4.8	https://www.ebi.ac.uk/arrayexpress/experiments/E-GEOD-22688/
	GEOD38961	3	1 (33%)	2 (66%)	35.7 ±14.2	https://www.ebi.ac.uk/arrayexpress/experiments/E-GEOD-38961/
**PBMCs** (whole blood derived)	GEOD49641	18	10 (56%)	1 (0.06%)	60.4 ±10.5	https://www.ebi.ac.uk/arrayexpress/experiments/E-GEOD-49641/
	GEOD17114	14	7 (50%)	9 (64%)	36.7 ±13.5	https://www.ebi.ac.uk/arrayexpress/experiments/E-GEOD-17114/
	GEOD6645	4	4 (100%)	0 (0%)	58.3 ±3.1	https://www.ncbi.nlm.nih.gov/geo/query/acc.cgi?acc=GSE66465
	MEXP1635	9	0 (0%)	5 (55%)	43.1 ±14.9	https://www.ebi.ac.uk/arrayexpress/experiments/E-MEXP-1635/
	GEOD38958	35	24 (69%)	0 (0%)	69.3 ±9.3	https://www.ebi.ac.uk/arrayexpress/experiments/E-GEOD-38958/
	EMTAB6531	4	1 (25%)	0 (0%)	55.3 ±2.5	https://www.ebi.ac.uk/arrayexpress/experiments/E-MTAB-6531/
**Nasal** (scrapings/ brushings)	GEOD11348	31	11 (35%)	31 (100%)	21.0 ±1.5	https://www.ebi.ac.uk/arrayexpress/experiments/E-GEOD-11348/
	EMATB4015	54	32 (59%)	0 (0%)	55.5 ±7	https://www.ebi.ac.uk/arrayexpress/experiments/E-MTAB-4015/
	GEOD16008	26	11 (42%)	18 (69%)	33.5 ±10.6	https://www.ebi.ac.uk/arrayexpress/experiments/E-GEOD-16008/
**Bronchial** (brushings	GEOD16008	26	11 (42%)	18 (69%)	33.8 ±10.4	https://www.ebi.ac.uk/arrayexpress/experiments/E-GEOD-16008/
	GEOD19667	48	NA	26 (54%)	39.1 ±13.6	https://www.ebi.ac.uk/arrayexpress/experiments/E-GEOD-19667/
